# Polygenic Risk Is Associated With Long-Term Coronary Plaque Progression and High-Risk Plaque

**DOI:** 10.1016/j.jcmg.2024.06.015

**Published:** 2024-08-14

**Authors:** Nick S. Nurmohamed, Injeong Shim, Emilie L. Gaillard, Shirin Ibrahim, Michiel J. Bom, James P. Earls, James K. Min, R. Nils Planken, Andrew D. Choi, Pradeep Natarajan, Erik S.G. Stroes, Paul Knaapen, Laurens F. Reeskamp, Akl C. Fahed

**Affiliations:** a Department of Cardiology, Amsterdam Cardiovascular Sciences, Amsterdam UMC, Vrije Universiteit Amsterdam, Amsterdam, the Netherlands;; b Department of Vascular Medicine, Amsterdam Cardiovascular Sciences, Amsterdam UMC, University of Amsterdam, Amsterdam, the Netherlands;; c Division of Cardiology, The George Washington University School of Medicine, Washington, DC, USA;; d Cardiovascular Disease Initiative, Broad Institute of MIT and Harvard, Cambridge, Massachusetts, USA;; e Department of Digital Health, Samsung Advanced Institute for Health Sciences and Technology, Sungkyunkwan University, Samsung Medical Center, Seoul, Republic of South Korea;; f Cleerly Inc, Denver, Colorado, USA;; g Department of Radiology and Nuclear Medicine, Amsterdam UMC, Universiteit van Amsterdam, Amsterdam, the Netherlands;; h Cardiovascular Research Center, Department of Medicine, Massachusetts General Hospital, Harvard Medical School, Boston, Massachusetts, USA.

**Keywords:** atherosclerosis, atherosclerotic cardiovascular disease, atherosclerosis imaging, coronary artery disease, coronary CT angiography, polygenic risk score, quantitative computed tomography

## Abstract

**BACKGROUND:**

The longitudinal relation between coronary artery disease (CAD) polygenic risk score (PRS) and long-term plaque progression and high-risk plaque (HRP) features is unknown.

**OBJECTIVES:**

The goal of this study was to investigate the impact of CAD PRS on long-term coronary plaque progression and HRP.

**METHODS:**

Patients underwent CAD PRS measurement and prospective serial coronary computed tomography angiography (CTA) imaging. Coronary CTA scans were analyzed with a previously validated artificial intelligence–based algorithm (atherosclerosis imaging–quantitative computed tomography imaging). The relationship between CAD PRS and change in percent atheroma volume (PAV), percent noncalcified plaque progression, and HRP prevalence was investigated in linear mixed-effect models adjusted for baseline plaque volume and conventional risk factors.

**RESULTS:**

A total of 288 subjects (mean age 58 ± 7 years; 60% male) were included in this study with a median scan interval of 10.2 years. At baseline, patients with a high CAD PRS had a more than 5-fold higher PAV than those with a low CAD PRS (10.4% vs 1.9%; *P* < 0.001). Per 10 years of follow-up, a 1 SD increase in CAD PRS was associated with a 0.69% increase in PAV progression in the multivariable adjusted model. CAD PRS provided additional discriminatory benefit for above-median noncalcified plaque progression during follow-up when added to a model with conventional risk factors (AUC: 0.73 vs 0.69; *P* = 0.039). Patients with high CAD PRS had an OR of 2.85 (95% CI: 1.14–7.14; *P* = 0.026) and 6.16 (95% CI: 2.55–14.91; *P* < 0.001) for having HRP at baseline and follow-up compared with those with low CAD PRS.

**CONCLUSIONS:**

Polygenic risk is strongly associated with future long-term plaque progression and HRP in patients suspected of having CAD.

## CENTRAL ILLUSTRATION

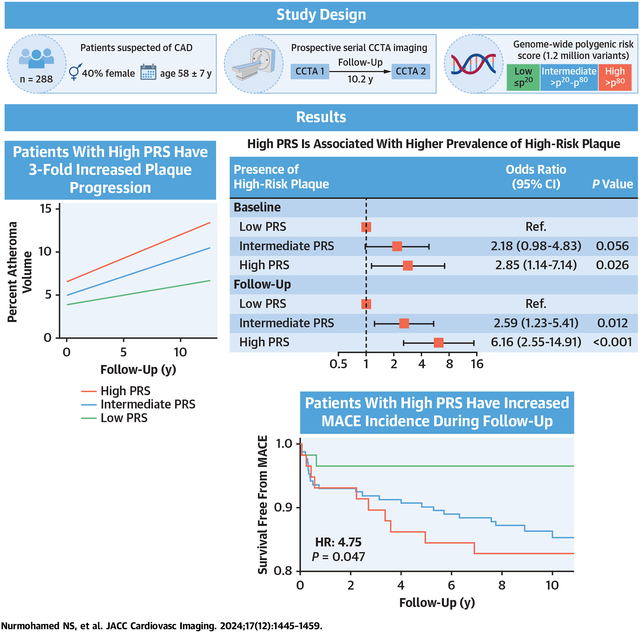

p20 and p80 refer to the 20th and 80th percentile of the polygenic risk score. CAD = coronary artery disease; CCTA = coronary CT angiography; MACE = major adverse cardiovascular events; PRS = polygenic risk score for coronary artery disease; Ref. = Reference.

Genomic risk of coronary artery disease (CAD), as measured by polygenic risk scores (PRS), has emerged as a potential clinical tool to identify patients who might be “flying under the radar” earlier in life.^1–4^ About 40% to 60% of CAD risk is attributed to heritability and is driven by many variants across the genome, each imparting a minimal risk increase or decrease.^5^ A CAD PRS summarizes the total number of genetic variants and their associated CAD risk for an individual in a single number.^1,2,6^ Although conventional cardiovascular prevention paradigms have focused on clinical risk factors with a relatively large impact such as hypercholesterolemia or hypertension, several studies have shown that incorporating CAD PRS adds to the performance of clinical risk calculators and improves the precision of risk stratification.^3,7–9^ Because DNA information is present from time of birth and represents lifetime exposure, CAD PRS could quantify risk before the onset of clinical risk factors,^3,4,10^ even in late middle–aged individuals or those with previous cardiovascular events.^11^ Importantly, post hoc analyses of clinical trial data have also shown that individuals with high CAD PRS experience disproportionately increased benefit from lipid-lowering therapy.^12–15^

Coronary computed tomography angiography (CTA) enables high-resolution quantification and characterization of plaque. Percent atheroma volume (PAV), presence of high-risk plaque (HRP) characteristics, and progression of plaque over time have all been associated with cardiovascular outcomes.^16–20^ As such, coronary CTA is emerging as a powerful tool to evaluate subclinical coronary atherosclerosis and to test novel interventions for CAD, providing early efficacy evidence and mechanistic insights.

A CAD PRS is traditionally developed and trained on clinical endpoints such as myocardial infarction and coronary revascularizations, which resemble end-stage disease manifestations of the underlying pathophysiological process of coronary atherosclerosis.^3,21,22^ It is, however, not fully understood how the coronary plaque burden and/or phenotype is modified by an individual’s genetic background. Several cross-sectional studies have shown that patients with a high CAD PRS have a higher coronary plaque burden.^23–25^ The longitudinal relationship between CAD PRS and long-term plaque progression and HRP characteristics has not been unraveled to date, however.

The current study aimed to investigate the effect of CAD PRS on long-term coronary plaque progression and HRP using a prospective cohort of 288 patients undergoing genotyping and serial coronary CTA imaging with a scan interval of 10 years.

## METHODS

### PATIENT POPULATION.

This prospective long-term serial coronary CTA study was performed in a cohort of patients who underwent baseline coronary CTA imaging for suspected stable CAD between 2008 and 2014 at the Amsterdam University Medical Centers (Amsterdam, the Netherlands).^20,26^ At time of baseline imaging, patients had no history of CAD. Per the research protocol, patients were invited for repeat coronary CTA imaging after 10 years for a follow-up study, regardless of symptoms or history. The study complied with the Declaration of Helsinki. The current follow-up study was separately approved by the local ethics committee, and participants provided separate informed consent for the follow-up coronary CTA study.

A total of 299 of 539 patients undergoing baseline coronary CTA underwent repeat coronary CTA imaging, of whom 288 provided informed consent for DNA analysis and had a plasma sample available (Supplemental Figure 1).

### GENOTYPING PRS CALCULATION.

DNA was genotyped by using a customized Global Screening Array v3 genotype array (Illumina) (Supplemental Methods).^27^ A recently published multiancestry CAD PRS (GPS_Mult_), consisting of 1.2 million common DNA variants, was calculated in all individuals.^3^ A low, intermediate, and high polygenic score for CAD was defined as a polygenic score in the first quintile (low), second to fourth quintile (intermediate), and fifth quintile (high), respectively, using cohort-specific cutoffs as performed previously.^12,28,29^

### CORONARY CTA IMAGING.

At baseline imaging, all patients underwent combined coronary artery calcium scoring (CACS) and coronary CTA using ≥64 slice coronary CTA scanners from the same manufacturer (Philips Healthcare), as described previously (Supplemental Methods).^20^ At follow-up, patients also underwent combined CACS and coronary CTA using a third-generation dual source CT scanner (SOMATOM Force, Siemens Healthineers).

### ATHEROSCLEROSIS IMAGING–QUANTITATIVE COMPUTED TOMOGRAPHY ANALYSIS.

An artificial intelligence–based software approach was used to analyze the coronary CTA images (atherosclerosis imaging–quantitative computed tomography) (Cleerly Inc) (Supplemental Methods).^30^ Coronary plaque volume was normalized to vessel volume, calculated as: plaque volume/vessel volume × 100%. These normalized volumes were reported as PAV, percent noncalcified plaque volume, and percent calcified plaque volume. Arterial remodeling was calculated by examining the lesion diameter divided by the normal reference diameter. Positive remodeling (PR) was defined as a ratio ≥1.1. HRPs were defined as coronary lesions with presence of both low-density noncalcified plaque and PR. The Coronary Artery Disease Reporting and Data System 2.0 was used to grade luminal stenosis.^31^

A total of 479 vessels were uninterpretable due to impaired image quality (motion, poor opacification, beam hardening, or other artifact), and 92 vessels were uninterpretable at follow-up due to stent placement, resulting in a total of 571 (10.7%) uninterpretable vessels. Uninterpretable vessels were excluded at both the baseline and follow-up imaging analyses to ensure a 1-to-1 comparison.

### STUDY OUTCOMES.

Outcomes were defined as the absolute change in PAV and percent noncalcified plaque volume, which was calculated subtracting baseline values from follow-up values. Secondary outcomes were defined as change in calcified plaque volume, as well as presence of low-density plaque and HRP. Lastly, a composite major adverse cardiovascular events (MACE) outcome of nonfatal myocardial infarction, nonfatal stroke, and coronary revascularization during follow-up was used to investigate the relationship between CAD PRS, PAV, and MACE. Events were adjudicated as described previously, and early revascularizations as a result from the baseline imaging were excluded from the composite outcome.^20^

### STATISTICAL ANALYSIS.

The relationship between the continuous PRS as well as PRS groups (low, intermediate, and high) and plaque volumes at baseline was assessed by using linear regression models with the plaque volumes as dependent variables on a perpatient level. On the per-vessel level, identical variables were used in linear mixed-effect regression models with within-patient clustering of vessels. The relationship between continuous PRS as well as PRS groups (low, intermediate, and high) and plaque volumes over time was assessed by using linear mixed-effect regression models with random intercept to account for repeated measures on a perpatient level with additional clustering to account for the different vessels in the per-vessel models. In these models, an interaction term between time and the different PRS groups was included to assess the association between PRS and plaque progression. The difference in plaque volumes and change in plaque volumes was graphically displayed over time using the estimates from the univariate linear mixed models for the different PRS groups (ie, low, intermediate, or high PRS). The relationship between the continuous PRS as well as PRS groups and HRP was assessed using logistic regression models, separate for baseline and follow-up imaging. The effect of PRS and statin use on noncalcified plaque regression (noncalcified plaque volume change <0) was also assessed using logistic regression.

The event-free survival during follow-up in patients with low, intermediate, and high PRS was shown using a Kaplan-Meier analysis, and HRs were calculated from a multivariable Cox regression model. A similar approach was used dividing patients into above- and below-median PAV and PRS. All models were adjusted for the first 5 principal components for genetic ancestry. The multivariable linear regression, linear mixed-effects, logistic regression, and Cox regression models were additionally adjusted for age, sex, and conventional risk factors (systolic blood pressure, low-density lipoprotein [LDL] cholesterol, high-density lipoprotein cholesterol, triglycerides, diabetes mellitus type 2 [yes/no], body mass index, history of smoking [yes/no], family history of CAD [yes/no], and statin intensity at baseline [low/moderate/high]), as well as coronary artery bypass grafting during follow-up and statin intensity at follow-up for the plaque progression analyses. The discriminatory value of a model with conventional risk factors was compared to models with conventional risk factors and baseline plaque volumes as well as PRS in an area under the curve (AUC) analysis with DeLong 95% CIs for prediction of above-median PAV progression.

Data are presented as mean ± SD for normally distributed variables and as median (Q1-Q3) for nonnormally distributed data. The normality of data distribution was assessed by using histograms and probability plots. Categorical variables are expressed as absolute numbers and percentages. Independent sample Student’s *t*-tests, Wilcoxon tests, Mann-Whitney U tests, and Kruskal-Wallis tests were used where appropriate. All statistical analyses were performed by using Posit PBC software version 4.0.3 (R Foundation).

## RESULTS

### PATIENT CHARACTERISTICS.

The 288 subjects had a mean age at baseline of 58 ± 7 years, and 174 (60%) were male. The ethnicity of the patients was predominantly White European (252 [87.5%]); 17 (5.9%) were Asian, 8 (2.8%) were of African descent, 8 (2.8%) were of Middle Eastern or North African descent, and 3 (1.0%) patients had a different or mixed ethnicity. Subjects experienced a range of symptoms comprising typical angina (91 [32%]), atypical angina (105 [37%]), and nonspecific chest pain (88 [31%]) as reason for referral for baseline coronary CTA imaging. Baseline characteristics were generally similar between the low, intermediate, and high PRS groups, except for LDL cholesterol and use of statins (Table 1). At baseline, median PAV was 4.9% (Q1-Q3: 1.1%−13.0%), reflecting a total plaque volume of 126.3 mm^3^ (Q1-Q3: 30.48–351.1 mm^3^). The median calcified plaque volume was 0.8% (Q1-Q3: 0.0%−4.0%) and 21.7 mm^3^ (Q1-Q3: 0.0–105.6 mm^3^), whereas noncalcified plaque volume was 3.4% (Q1-Q3: 1.1%−8.2%) and 88.6 mm^3^ (Q1-Q3: 27.4–233.4 mm^3^) (Table 2).

### CAD PRS IS ASSOCIATED WITH BASELINE CORONARY PLAQUE BURDEN.

At baseline, PRS was associated with coronary plaque volumes. Patients with a high PRS (ie, >80th percentile) had a >5-fold higher PAV compared with those with a low PRS (10.4 [Q1-Q3: 3.2–18.9] vs 1.9 [Q1-Q3: 0.5–8.1]; *P* < 0.001) (Table 2). Percent noncalcified plaque volume was also higher in those with a high PRS (6.4 [Q1-Q3: 2.0–11.6]) than in patients with a low PRS (1.7 [Q1-Q3: 0.5–4.9]; *P* < 0.001). Patients with a high PRS also had a higher calcified plaque volume at baseline (2.8 [Q1-Q3: 0.7–7.5]) compared with patients with low PRS (0.1 [Q1-Q3: 0.0–2.9]; *P* < 0.001), in whom calcified plaque volume was almost negligible.

After adjustment for age, sex, genetic ancestry, and conventional risk factors, every 1 SD increase in PRS was associated with a 0.97% (95% CI: −0.07 to 2.00; *P* = 0.069) higher PAV, a 0.43% (95% CI: −0.19 to 1.05; *P* = 0.176) higher noncalcified plaque volume, and a 0.55% (95% CI: 0.03–1.07; *P* = 0.038) higher calcified plaque volume at baseline (Table 3). Patients with a high PRS had a 3.31% higher PAV (95% CI: 0.06–6.56; *P* = 0.046), a 1.58% (95% CI: −0.37 to 3.54; *P* = 0.114) higher percent noncalcified plaque volume, and a 1.79% (95% CI: 0.15–3.42; *P* = 0.033) higher percent calcified plaque volume than those with low PRS at baseline. The baseline findings were similar in the sensitivity analysis on a per-vessel level (Supplemental Table 1).

### CAD PRS IS ASSOCIATED WITH CORONARY PLAQUE PROGRESSION, INDEPENDENT OF AGE, SEX, AND CONVENTIONAL RISK FACTORS.

We then sought to determine if plaque progression varied according to genetic risk. The median interval between baseline and follow-up imaging was 10.2 years (Q1-Q3: 8.7–11.2 years). Patients with a high PRS had the highest progression of PAV, 5.05% (Q1-Q3: 2.06%−9.44%), which was higher than 1.28% (Q1-Q3: 0.37%−3.86%) for patients with low PRS (*P* = 0.001) (Table 4). Similar trends were observed for change in noncalcified plaque and calcified plaque over the 10-year period and were directionally similar after adjustment for baseline plaque volumes (Supplemental Table 2).

**FIGURE 1:**
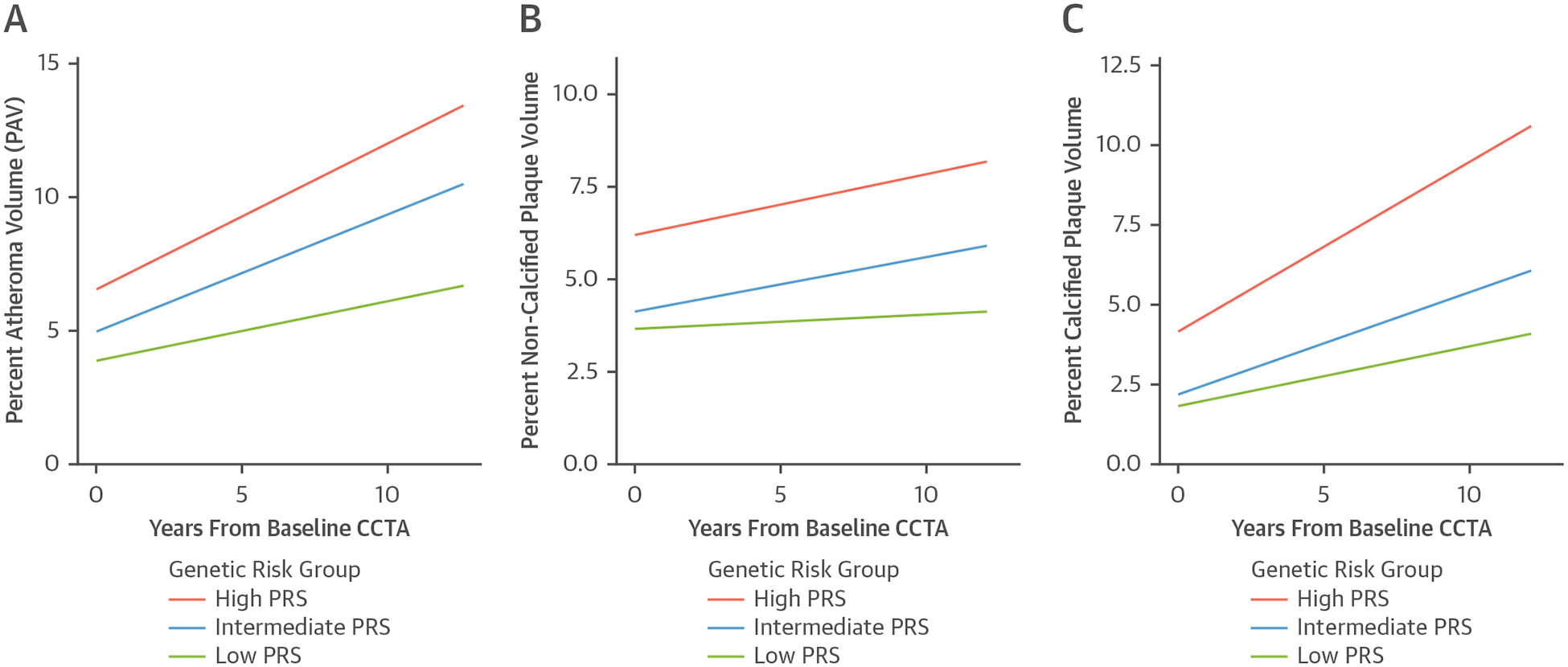
Association of CAD PRS With Plaque Progression Over Long-Term Follow-Up Shown are estimates from the unadjusted linear mixed-effect models for the different genetic risk groups in this study for percent atheroma volume (PAV) (A), percent noncalcified plaque volume (B), and percent calcified plaque volume (C). CAD = coronary artery disease; CCTA = coronary computed tomography angiography; PRS = polygenic risk score.

CAD PRS remained strongly associated with the rate of plaque progression over the 10-year follow-up period after adjustment for age, sex, genetic ancestry, conventional risk factors, and baseline plaque volume (Figure 1, Table 5). Every 1 SD increase in PRS was associated with a 0.69% (95% CI: 0.12–1.27; *P* = 0.019) increase in PAV. Compared vs patients with a low PRS, patients with a high PRS had 2.75% (95% CI: 0.91−4.58; *P* = 0.004) higher PAV progression. The accelerated progression in plaque with increasing genetic risk was observed for both calcified and noncalcified plaque. Compared vs patients with a low PRS, patients with a high PRS had a 1.27% (95% CI: 0.13–2.41; *P* = 0.030) higher noncalcified plaque volume progression and a 2.07% (95% CI: 0.53–3.61; *P* = 0.009) higher calcified plaque volume progression. Conversely, PRS was inversely associated with regression of noncalcified plaque (OR: 0.72 [95% CI: 0.54–0.96] per 1 SD increase in PRS; *P* = 0.027), although there was no association between statin use and noncalcified plaque regression (OR: 1.04 [95% CI: 0.81–1.34]; *P* = 0.76).

The plaque progression findings were similar in the sensitivity analysis on a per-vessel level in the full patient population (Supplemental Table 3) and when restricted to patients using statins during follow-up (Supplemental Table 4).

Finally, the additional discriminatory value of PRS and baseline plaque beyond conventional risk factors for prediction of above-median PAV progression (>2.83%) and noncalcified plaque progression (>0.66%) was evaluated. For PAV progression, the model with conventional risk factors achieved an AUC of 0.74 (95% CI: 0.68–0.79). After addition of baseline PAV, the AUC increased to 0.83 (95% CI: 0.78–0.87) (Table 6). After the addition of PRS, the AUC increased further to 0.86 (95% CI: 0.81–0.90). For noncalcified plaque progression, the conventional model achieved an AUC of 0.68 (95% CI: 0.62–0.74), which did not meaningfully increase after addition of baseline plaque (AUC: 0.69; 95% CI: 0.63–0.75). After further addition of PRS, the AUC increased to 0.73 (95% CI: 0.67–0.79).

### CAD PRS IS ASSOCIATED WITH AN HRP PHENOTYPE.

**FIGURE 2:**
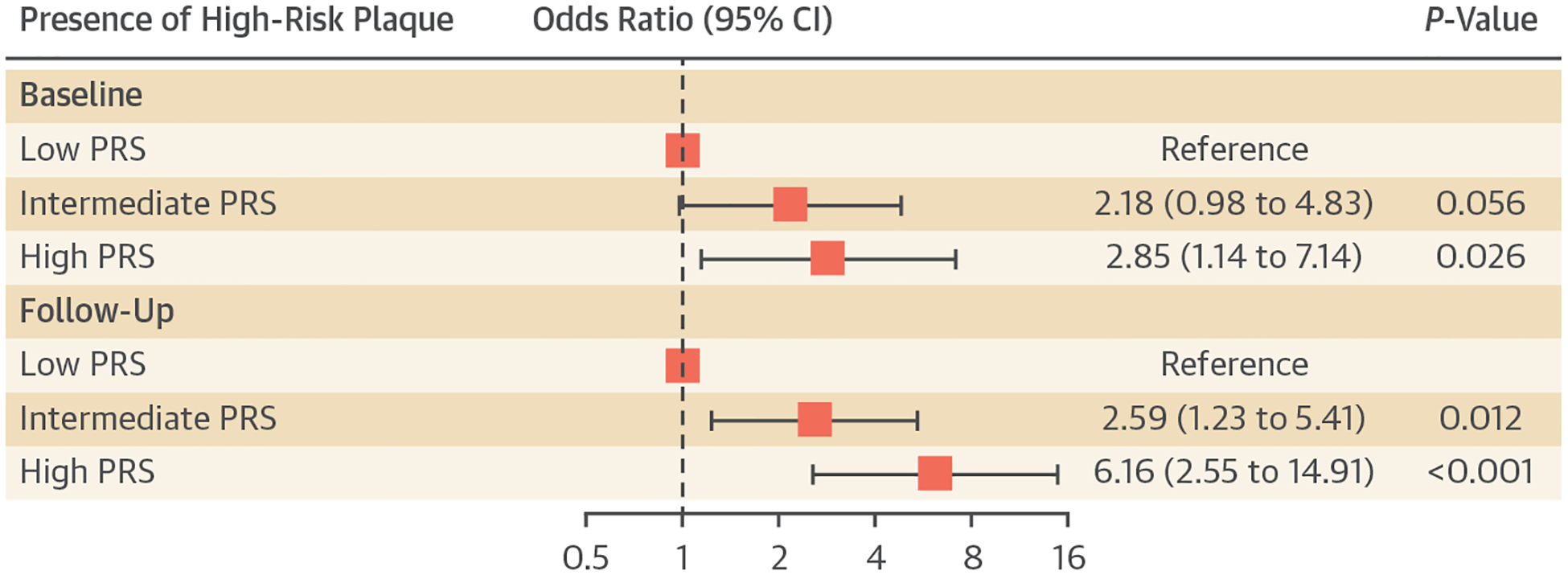
Association of CAD PRS With HRP Shown are adjusted ORs from logistic regression models for the presence of high-risk plaque (HRP) at baseline and follow-up imaging. Models were adjusted for age, sex, genetic ancestry, and conventional risk factors. Abbreviations as in Figure 1.

Because cardiovascular outcomes are predicted by HRP in addition to plaque volume itself, we next tested whether CAD PRS was associated with HRP at baseline and after the 10 years of follow-up. Patients with high and intermediate PRS had a higher risk at baseline and follow-up of having HRP (Figure 2). Adjusted for age, sex, genetic ancestry, and conventional risk factors, every 1 SD increase in PRS resulted in an OR of 1.41 (95% CI: 1.04–1.91; *P* = 0.028) for having HRP at baseline and an OR of 1.69 (95% CI: 1.26–2.27; *P* < 0.001) for having HRP at follow-up. Compared vs those with a low PRS, patients with a high PRS had an OR of 2.85 (95% CI: 1.14–7.14; *P* = 0.026) for having HRP at baseline and 6.16 (95% CI: 2.55–14.91; *P* < 0.001) for having HRP at follow-up.

**FIGURE 3:**
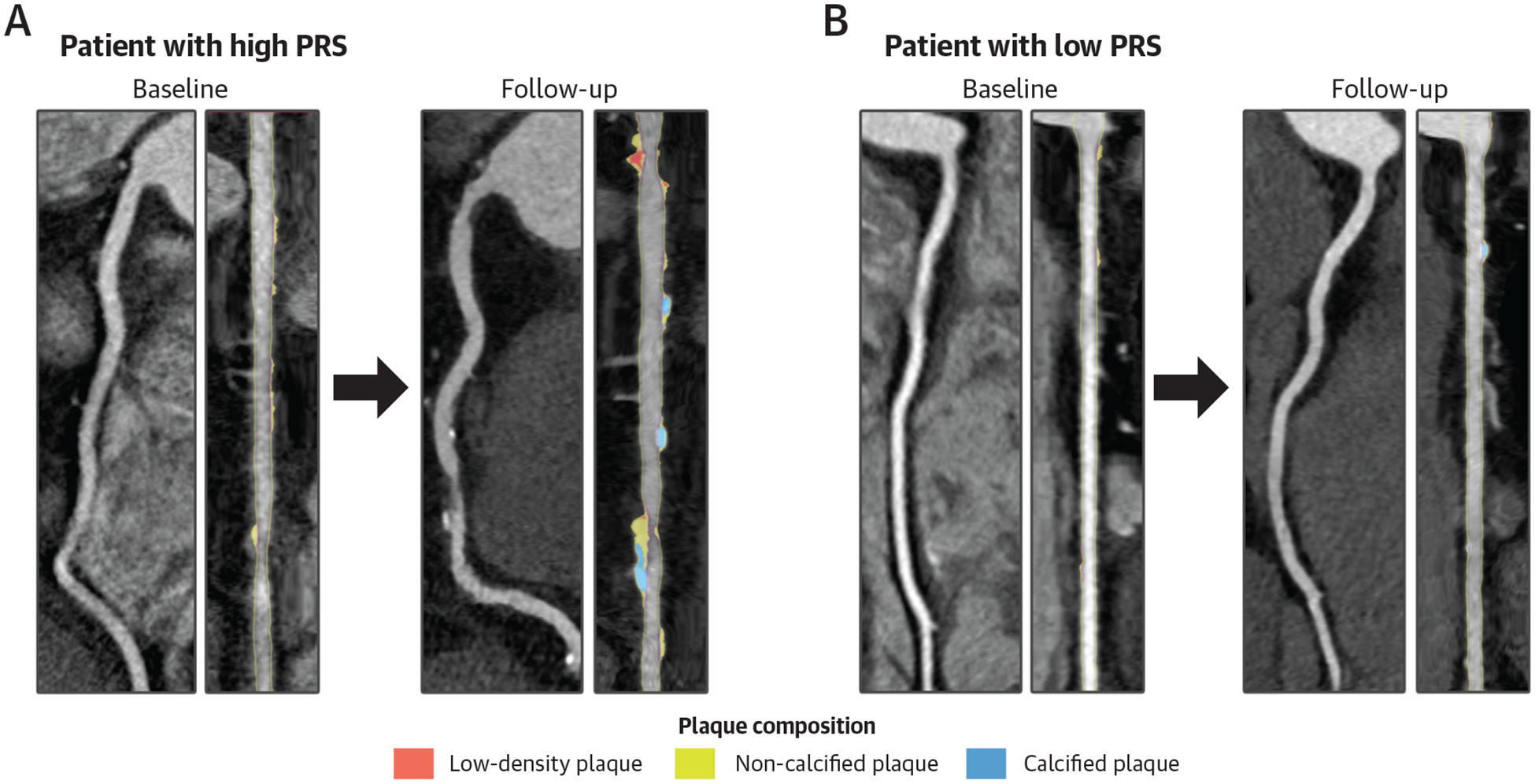
Coronary Plaque Progression and HRP in Patients With High and Low Polygenic Risk Shown are 2 case examples of a patient with high (A) and low (B) PRS. Shown are baseline coronary CTA curved reformatted (left) and straightened multiplanar reformatted (right) reconstructions of the right coronary artery. At baseline, patient A (high CAD PRS, 98th percentile) had a PAV of 16.5%, a percent noncalcified plaque volume (NCPV) of 6.4%, and a percent calcified plaque volume (CPV) of 10.0%, with no presence of HRP. During the 10-year follow-up, the plaque volumes for PAV, percent NCPV, and percent CPV progressed to 26.1%, 11.5%, and 14.6% at follow-up (58% PAV increase, 80% percent NCPV increase, and 46% percent CPV increase), respectively, indicative of important plaque progression. Furthermore, 2 HRPs with low-density plaque had developed. Patient B (low CAD PRS, 10th percentile) had a baseline PAV of 5.8%, a percent NCPV of 4.5%, and a percent CPV of 1.3%. During the 10-year follow-up, the plaque volumes for PAV, NCPV, and CPV changed to 7.6%, 4.9%, and 2.5% (31% PAV increase, 8% NCPV decrease, and 92% CPV increase), indicative of relative plaque stabilization. No HRPs developed throughout the follow-up. Abbreviations as in Figure 1.

Two case examples (high and low CAD PRS) of plaque progression and HRP are presented in Figure 3.

### GENOME-WIDE POLYGENIC SCORE IS ASSOCIATED WITH CARDIOVASCULAR EVENTS DURING 10-YEAR FOLLOW-UP.

**FIGURE 4:**
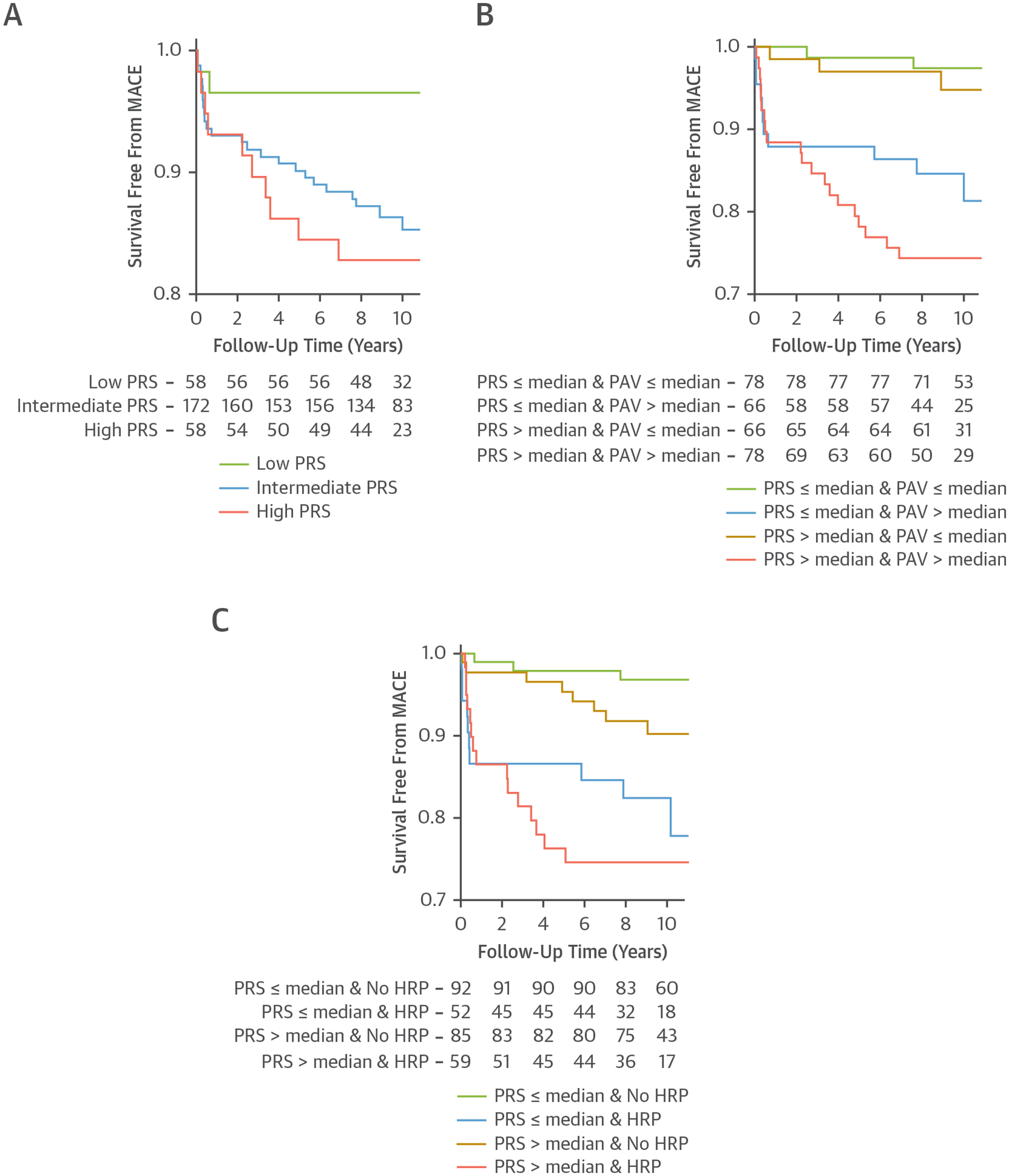
MACE Over Long-Term Follow-Up According to Genetic Risk Groups and Baseline Plaque Shown are 3 curves from Kaplan-Meier analyses for survival free from major adverse cardiovascular events (MACE), defined as all-cause mortality, nonfatal myocardial infarction, nonfatal stroke, and coronary revascularization. (A) MACE-free survival for the 3 PRS groups. (B) MACE-free survival in 4 groups divided based on PRS (above/below median) and baseline PAV (above/below median). (C) MACE-free survival in 4 groups divided based on PRS (above/below median) and presence of HRP. Abbreviations as in Figures 1 to 3.

During the median follow-up of 10.2 years, 37 (12.8%) of 288 patients experienced the composite MACE outcome. Twelve (4.2%) patients had a nonfatal myocardial infarction, 6 (2.1%) had a nonfatal stroke, and 19 (6.6%) underwent coronary revascularization. Patients with a higher CAD PRS had reduced survival free of MACE (Figure 4A). In the multivariable adjusted survival analysis, patients with a high PRS and intermediate PRS had an HR of 4.75 (95% CI: 1.02–22.17; *P* = 0.047) and 4.58 (95% CI: 1.06–19.78; *P* = 0.042), respectively, for the composite MACE outcome. When PRS and baseline PAV were combined into one model, only PAV remained independently associated with MACE (Figure 4B). After multivariable adjustment, patients with a PRS above the median had a HR of 1.98 (95% CI: 0.98–4.03; *P* = 0.059) for MACE compared with patients with below-median PRS, whereas PAV above the median was associated with an HR of 6.63 (95% CI: 2.41–18.27; *P* < 0.001). Similarly, when PRS was combined with the presence of HRP, only HRP remained independently associated with MACE (Figure 4C). After multivariable adjustment, patients with a PRS above the median had an HR of 1.95 (95% CI: 0.96–3.94; *P* = 0.065) for MACE compared with patients with below-median PRS, whereas the presence of HRP was associated with an HR of 3.72 (95% CI: 1.66–8.35; *P* = 0.001).

## DISCUSSION

Leveraging a PRS and serial quantitative coronary CTA imaging data with a 10-year interval in 288 patients, this study shows the important role of CAD PRS in the progression of coronary plaque over long-term follow-up. In patients undergoing coronary CTA for suspected CAD, CAD PRS was predominantly associated with increased noncalcified plaque volume at baseline. During >10 years of follow-up, the full spectrum of coronary plaque increased almost 3-fold as much in those with a high PRS compared with those with a low PRS. In addition, both at baseline and follow-up imaging, patients with high CAD PRS had an up to 7-fold increased risk of having high-risk coronary plaque. Patients with high PRS, especially those with above-average plaque volume burden or HRP at baseline, were at markedly increased risk of MACE during the long-term follow-up. Collectively, these data illustrate the impact of an individual’s genetic background on the development and progression of coronary artery plaque burden throughout life (Central Illustration).

To our knowledge, this study is the first to investigate the relationship between CAD PRS and long-term coronary plaque progression analyzed by use of serial coronary CTA imaging. Over the long term, patients with a high PRS had a >6-fold increase in noncalcified plaque volume progression compared with individuals with a low PRS. This markedly accelerated plaque progression persisted even after adjustment for conventional risk factors and baseline plaque volume. Moreover, PRS provided important additional discriminatory value to identify those with above-median plaque progression over prediction with conventional factors only. The prognostic value of the PRS for plaque progression but also MACE beyond conventional risk scoring observed in the current study is likely due to the capture of lifelong exposure to genetic risk by PRS as opposed to conventional risk factors, which generally develop much later in life. In addition, the PRS likely encompasses a complex interplay of a multitude of atherogenic pathways that cannot be captured by measuring a handful of conventional risk factors.

Previous studies have investigated the association between PRS and CACS as well as qualitatively assessed coronary CTA. In a post hoc analysis in 605 participants from the PROMISE (Prospective Multicenter Imaging Study for Evaluation of Chest Pain) trial,^23^ it was shown that those in the highest quintile of PRS had a 5 times higher risk of having obstructive CAD. Christiansen et al^24^ reported that every SD increase in PRS was associated with a 78% higher CACS (*P* < 0.001) and the presence of obstructive plaque (OR: 1.78; *P* < 0.001). Recently, an autopsy study of 954 cases found that subjects in the highest PRS quintile had more obstructive stenosis, severe atherosclerosis (OR: 3.77 [95% CI: 2.10–6.78]; *P* < 0.001), and plaque rupture (OR: 4.05 [95% CI: 2.26–7.24]; *P* < 0.001) compared with the lowest quintile after adjustment for clinical CAD risk factors.^25^

The current study is, to our knowledge, the first to investigate the relationship between PRS and quantitatively and serially assessed coronary CTA, and it adds to findings of prior cross-sectional studies. Due to the longitudinal nature of the prospective coronary CTA imaging with a unique interval that exceeded 10 years, we were able to estimate the effect of PRS on the long-term progression of coronary atherosclerosis. Furthermore, we used artificial intelligence–guided quantitative coronary CTA analysis, which has shown superior diagnostic accuracy for atherosclerosis and stenosis compared with visual/semiquantitative analysis in prior studies and allows for detailed plaque characterization and identification of HRP.^20,30^

Beyond the observed cross-sectional relationship between PRS and coronary plaque burden, importantly, the current data show that patients with higher PRS had increased plaque progression in a model, including baseline plaque volume during a 10-year follow-up. In fact, the average rate of plaque progression, adjusted for conventional risk factors and plaque volume at baseline, was 3-fold higher in patients with a high PRS compared with those with a low PRS. These findings imply that, despite receiving medical and interventional treatment for coronary CTA–defined plaque burden in the current nonblinded study, patients with a higher PRS still have profound plaque progression, in contrast to patients with a low PRS. Furthermore, in the events analysis, those with both a high PRS and high PAV or HRP were at the highest risk for MACE during follow-up, although PRS was not an independent predictor after multivariable adjustment, including plaque burden. This may imply that a high PRS can further accelerate the development of rupture-prone plaques in patients with an already significant plaque burden. This is consistent with recent finding that CAD PRS is predictive of recurrent CAD events even in patients with established CAD.^11^ Therefore, assessing PRS in patients undergoing risk stratification with coronary CTA may improve identification of those at the highest risk of MACE.

On the contrary, a recent analysis by Khan et al^32^ in the MESA (Multi-Ethnic Study of Atherosclerosis) and Rotterdam studies showed no additional benefit of PRS to risk stratification with clinical risk factors, whereas CACS did provide prognostic benefit. However, there were 2 important differences with the current study. First, both the MESA and Rotterdam study were population based, while the current study recruited patients suspected of having CAD. Second, the participants from the Rotterdam study, although from a Dutch population with a similar life expectancy, were >10 years older at baseline than the patients in the current study (67.6 years vs 57.5 years), while polygenic risk has been suggested of prognostic value in predominantly younger adults. During this 10-year time frame, the patients with a high PRS in the current study displayed important progression of calcified plaque, which might explain the additional prognostic value of CACS found in the study by Khan et al.^32^ Studies launched over the past year are prospectively evaluating whether a subclinical approach to prevention of CAD leveraging CAD PRS enrichment and/or coronary CTA could be superior to our conventional risk-based approach (NCT03920176, NCT06112418, NCT05819814, NCT05850091, and NCT05800093).

In addition to the relationship with noncalcified plaque at baseline, patients with a high PRS had an increased prevalence of HRP (defined as the simultaneous presence of low-density noncalcified plaque and positive remodeling) at both baseline and follow-up imaging, after adjustment for CAD risk factors. Although some prior studies have found a relatively weak relationship between PRS and a HRP, the current findings were consistent over more than 10 years of follow-up, reaffirming the high prevalence of HRP in those with increased genetic CAD risk. At baseline, patients with a high PRS had a more than 3-fold higher risk of having HRP, which increased to a >7-fold higher risk at follow-up. These high-risk, rupture-prone plaques are likely to play a major role in the observed increased incidence of MACE in those with a high PRS, as previously suggested by Cornelissen et al,^25^ who found at autopsy that patients with a higher PRS had a 4-fold higher odds of plaque rupture.

The impact of PRS on plaque progression and HRP in the current cohort of relatively young patients suspected of CAD has several implications for clinical practice. The notion of increased plaque progression in patients with high PRS, independent of baseline plaque burden and conventional risk factors, implies that measurement of PRS could identify patients at very high risk of disease progression who would be classified as low-risk individuals with conventional risk scoring or imaging. With the broad availability of conventional and novel atherosclerotic cardiovascular disease (ASCVD) risk-lowering therapies, including LDL cholesterol–lowering agents (statins, bempedoic acid, and proprotein convertase subtilisin-kexin type 9 inhibition), glucagon-like peptide-1 agonists, sodium-glucose cotransporter 2 inhibitors, anti-inflammatory therapies (colchicine), and omega-3 fatty acids, we have ample tools to lower risk in these individuals, once identified. Some of those therapies have already been proven to have a disproportionately increased benefit in individuals with a high PRS in post hoc analyses of clinical trials.^12–15^ Inclusion of PRS in ASCVD risk estimation holds promise to better identify those at increased lifetime risk of ASCVD.^33^

The current study illustrates that one additional impact of PRS may be in patients with a relatively high plaque burden, who are additionally predisposed to a sequel of complex genetically determined pathophysiological processes leading to rapid plaque progression, development of HRPs, and subsequent MACE. Overall, PRS may facilitate identification of individuals with adverse genetic predisposition long before they develop high-risk atherosclerotic lesions resulting in MACE.

### STUDY LIMITATIONS.

First, the current study was focused on patients with suspected symptomatic CAD. The association of CAD PRS with plaque progression in a subclinical asymptomatic population remains unknown. However, the current data support the concept that combining genetic risk with noninvasive plaque imaging might help us re-imagine how to prevent CAD, a concept that will be tested in the PROACT (Polygenic Risk-Based Detection of Subclinical Atherosclerosis) clinical trials (NCT05819814 and NCT05850091). Second, this was a single-center study with 288 subjects; however, the 2,888 patient-years of follow-up due to the median interval of 10 years between baseline and follow-up scans and quantitative analyses with continuous measures (both CAD PRS and plaque measures) provided appropriate power. It is worth noting that the power for the MACE analysis was limited and should be interpreted as an exploratory analysis. Third, due to the long interval between baseline and follow-up imaging, there were inevitable differences between scanner type and protocol, which may have affected the current findings. Although overall PAV is unlikely to be affected by difference in scanners,^34^ plaque composition might have been interpreted differently, despite adjustment for scanner type and settings in the plaque analysis. In addition, several patients underwent revascularization between baseline and follow-up imaging, which required exclusion of the revascularized vessels at both time points. Because vessels undergoing revascularization had greater plaque volumes, the magnitude of the association between PRS and plaque progression might be an underestimation. Fourth, baseline coronary CTA results may have affected medical treatment during the long-term follow-up, as patients with high PRS were more frequently using statin therapy, which might have attenuated the effect of PRS on plaque progression in this study. Finally, this study was focused on individuals of European ancestry due to its singlesite nature. Although multiancestry CAD PRS performs reasonably well in multiple ancestries outside of Europe, the performance in individuals of African ancestries is reduced.^3^

## CONCLUSIONS

Polygenic risk is strongly associated with coronary plaque burden, accelerated plaque progression, and HRP prevalence and formation over long-term follow-up. These findings underscore the role of CAD PRS in plaque progression and its contribution to cardiovascular events. They also emphasize the relevance of assessing both polygenic risk and presence of subclinical atherosclerosis with coronary CTA in patients suspected of having CAD.

## Supplementary Material

supplementary methods

APPENDIX For an expanded Methods section as well as supplemental tables, a figure, and references, please see the online version of this paper.

## Figures and Tables

**TABLE 1:** Baseline Characteristics According to Polygenic Risk Group

		Polygenic Risk			
	Overall (N = 288)	Low (n = 58)	Intermediate (n = 172)	High (n = 58)	*P* Value
Age, y	57.5 ± 7.3	57.9 ± 7.6	57.3 ± 7.4	57.5 ± 6.9	0.893
Male	174 (60)	30 (52)	106 (62)	38 (66)	0.277
Reason for referral					0.244
Nonspecific chest pain	88 (31)	16 (28)	58 (34)	14 (25)	
Atypical angina	105 (37)	27 (47)	54 (32)	24 (42)	
Typical angina	91 (32)	15 (26)	57 (34)	19 (33)	
Type 2 diabetes mellitus	47 (16)	7 (12)	27 (16)	13 (22)	0.302
Body mass index, kg/m^2^	27.2 ± 4.3	26.7 ± 4.4	27.0 ± 4.3	27.9 ± 3.9	0.118
History of smoking	82 (28)	21 (36)	45 (26)	16 (28)	0.459
Family history of CAD	160 (56)	28 (48)	103 (60)	29 (50)	0.194
Hypertension	122 (42)	20 (34)	72 (42)	30 (52)	0.167
Hypercholesterolemia	109 (38)	18 (31)	62 (36)	29 (50)	0.081
Statin use					0.012
No statin	106 (37)	27 (47)	67 (39)	12 (21)	
Low intensity	3 (1.0)	1 (1.7)	2 (1.2)	0 (0)	
Moderate intensity	146 (51)	23 (40)	89 (52)	34 (59)	
High intensity	33 (11)	7 (12)	14 (8.1)	12 (21)	
Beta-blocker use	175 (62)	33 (58)	107 (63)	35 (61)	0.794
Aspirin use	207 (73)	38 (67)	121 (71)	48 (84)	0.079
Calcium-channel blocker use	77 (27)	13 (23)	47 (28)	17 (30)	0.680
LDL cholesterol, mg/dL	101 ± 38	113 ± 40	97 ± 35	105 ± 40	0.056
LDL cholesterol, mg/dL, adjusted for statin use^a^	126.87 ± 48.00	136 ± 50	120 ± 44	139 ± 53	0.023
HDL cholesterol, mg/dL	56 ± 20	59 ± 19	55 ± 18	54 ± 23	0.301
Triglycerides, mg/dL	124 (89–188)	124 (89–151)	128 (89–195)	120 (88–210)	0.476
Total cholesterol, mg/dL	175 (62)	33 (58)	107 (63)	35 (61)	0.326
PRS percentile	50 (25–75)	10 (5–15)	50 (35–65)	90 (85–95)	

Values are mean ±SD, n (%), or median (Q1-Q3).

a Low-density lipoprotein (LDL) cholesterol was adjusted for statin use considering an average reduction of 30%.

CAD = coronary artery disease; HDL = high-density lipoprotein; PRS = polygenic risk score.

**TABLE 2:** Coronary Atherosclerosis Quantification at Baseline According to Polygenic Risk Group

		Polygenic Risk			
	Overall (N = 288)	Low (n = 58)	Intermediate (n = 172)	High (n = 58)	*P* Value
Total plaque volume, mm^3^	126.3 (30.48–351.1)	46.4 (12.4–197.4)	128.5 (29.2–330.9)	286.6 (86.6–530.7)	<0.001
Noncalcified plaque volume, mm^3^	88.6 (27.4–233.4)	43.4 (11.4–107.8)	94.3 (25.1–213.5)	187.0 (58.9–312.8)	<0.001
Calcified plaque volume, mm^3^	21.7 (0.0–105.6)	2.5 (0.0–63.9)	15.6 (0.0–92.5)	73.5 (18.6–225.8)	<0.001
Percent atheroma volume, %	4.9 (1.1–13.0)	1.9 (0.5–8.1)	4.7 (1.0–11.2)	10.4 (3.2–18.9)	<0.001
Percent noncalcified plaque volume, %	3.4 (1.1–8.2)	1.7 (0.5–4.9)	3.4 (1.0–7.9)	6.4 (2.0–11.6)	<0.001
Percent calcified plaque volume, %	0.8 (0.0–4.0)	0.1 (0.0–2.9)	0.7 (0.0–3.9)	2.8 (0.7–7.5)	<0.001
Presence of high-risk plaque	111 (39)	14 (24)	66 (38)	31 (53)	0.005
Presence of obstructive stenosis	104 (36)	10 (17)	64 (37)	30 (52)	0.001
CAD-RADS score					0.003
0	10 (3.5)	3 (5.2)	7 (4.1)	0 (0)	
1	130 (45)	35 (60)	77 (45)	18 (31)	
2	44 (15)	10 (17)	24 (14)	10 (17)	
3	38 (13)	4 (6.9)	19 (11)	15 (26)	
4 or 5	66 (23)	6 (10)	45 (26)	15 (26)	

Values are median (Q1-Q3) or n (%).

CAD-RADS = Coronary Artery Disease Reporting and Data System.

**TABLE 3:** Association of CAD PRS With Baseline Plaque Volumes

	Model 1 (Unadjusted)		Model 2 (Adjusted for Age, Sex, Genetic Ancestry, and Conventional Risk Factors)	
	Beta (95% CI)	*P* Value	Beta (95% CI)	*P* Value
Baseline percent atheroma volume				
CAD PRS, per 1 SD increase	1.22 (0.20–2.25)	0.020	0.97 (−0.07 to 2.00)	0.069
Genetic risk groups				
Low PRS	Ref.	Ref.	Ref.	Ref.
Intermediate PRS	0.51 (−2.14 to 3.16)	0.707	0.26 (−2.4 to 2.93)	0.846
High PRS	4.46 (1.24–7.68)	0.007	3.31 (0.06–6.56)	0.046
Baseline percent noncalcified plaque volume				
CAD PRS, per 1 SD increase	0.54 (−0.07 to 1.15)	0.086	0.43 (−0.19 to 1.05)	0.176
Genetic risk groups				
Low PRS	Ref.	Ref.	Ref.	Ref.
Intermediate PRS	0.18 (−1.40 to 1.76)	0.821	0.06 (−1.54 to 1.66)	0.939
High PRS	2.08 (0.15–4.00)	0.035	1.58 (−0.37 to 3.54)	0.114
Baseline percent calcified plaque volume				
CAD PRS, per 1 SD increase	0.70 (0.19–1.22)	0.008	0.55 (0.03–1.07)	0.038
Genetic risk groups				
Low PRS	Ref.	Ref.	Ref.	Ref.
Intermediate PRS	0.42 (−0.91 to 1.75)	0.493	0.31 (−1.03 to 1.65)	0.651
High PRS	2.44 (0.83–4.06)	<0.001	1.79 (0.15–3.42)	0.033

Linear regression models were created with percent atheroma volume, percent noncalcified plaque volume, and percent calcified plaque volume as dependent outcomes using both CAD PRS on a continuous scale and divided in the different genetic risk groups. The multivariable model was adjusted for age, sex, genetic ancestry, coronary revascularization, and conventional risk factors (hypertension, hypercholesterolemia, diabetes, body mass index, smoking status, family history of CAD, and statin use).

Ref. = Reference; other abbreviations as in Table 1.

**TABLE 4:** Changes in Plaque Volumes Between Baseline and 10-Year Follow-Up

		Polygenic Risk			
	Overall (N = 288)	Low (n = 58)	Intermediate (n = 172)	High (n = 58)	*P* Value
PAV					
Absolute change, %	2.83 (0.53–7.10)	1.28 (0.37–3.86)	2.73 (0.53–6.97)	5.05 (2.06–9.44)	0.001
Above-median progression		19 (33)	84 (49)	41 (71)	<0.001
Percent noncalcified plaque volume					
Absolute change, %	0.66 (0.00–2.55)	0.35 (−0.17to1.51)	0.67 (0.00–2.55)	2.10 (0.11–3.34)	0.010
Above-median progression		20 (34)	87 (51)	37 (64)	0.007
Percent calcified plaque volume					
Absolute change, %	1.21 (0.08–3.71)	0.42 (0.00–1.98)	1.21 (0.07–3.73)	1.96 (0.62–5.76)	0.002
Above-median progression		20 (34)	85 (49)	39 (67)	0.002

Values are median (Q1-Q3) or n (%). Shown is the absolute change in percent atheroma volumes (PAVs) for patients with low, intermediate, and high polygenic risk score (PRS) and the proportion of patients with above-median progression for PAV, noncalcified plaque volume, and calcified plaque volume. Vessels that were revascularized between baseline and follow-up were excluded from both baseline and follow-up plaque analysis.

**TABLE 5:** Association of CAD PRS With Plaque Progression Over 10 Years

	Model 1 (Unadjusted)		Model 2 (Adjusted for Age, Sex, Genetic Ancestry, Conventional Risk Factors, and Baseline Plaque Volume)	
	Beta (95% CI)	*P* Value	Beta (95% CI)	*P* Value
PAV, change per 10 years of follow-up				
CAD PRS, per 1 SD increase	1.12 (0.47–1.77)	<0.001	0.69 (0.12–1.27)	0.019
Genetic risk groups				
Low PRS	Ref.	Ref.	Ref.	Ref.
Intermediate PRS	1.95 (0.30–3.59)	0.021	1.11 (−0.36 to 2.58)	0.139
High PRS	4.35 (2.31–6.4)	<0.001	2.75 (0.91–4.58)	0.004
Percent noncalcified plaque volume, change per 10 years of follow-up				
CAD PRS, per 1 SD increase	0.35 (−0.02 to 0.71)	0.065	0.35 (−0.01 to 0.70)	0.058
Genetic risk groups				
Low PRS	Ref.	Ref.	Ref.	Ref.
Intermediate PRS	1.05 (0.11–1.98)	0.029	0.97 (0.06–1.89)	0.038
High PRS	1.22 (0.06–2.38)	0.040	1.27 (0.13–2.41)	0.030
Percent calcified plaque volume, change per 10 years of follow-up				
CAD PRS, per 1 SD increase	0.90 (0.35–1.45)	0.002	0.58 (0.09–1.06)	0.019
Genetic risk groups				
Low PRS	Ref.	Ref.	Ref.	Ref.
Intermediate PRS	1.28 (−0.12 to 2.69)	0.075	0.58 (−0.65 to 1.82)	0.354
High PRS	3.33 (2.07–5.66)	<0.001	2.07 (0.53–3.61)	0.009

Unadjusted and adjusted mixed effect models were constructed with PAV, percent noncalcified plaque volume, and percent calcified plaque volume as dependent outcomes. An interaction term between the duration of follow-up and CAD PRS (both continuous and the CAD PRS groups) and the other covariables was included to estimate plaque progression during follow-up. The multivariable model was adjusted for age, sex, genetic ancestry, coronary revascularization, and conventional risk factors (hypertension, hypercholesterolemia, diabetes, body mass index, smoking status, family history of CAD, and statin use).

Abbreviations as in Tables 1, 3, and 4.

**TABLE 6:** Discriminatory Value of Polygenic Risk, Baseline Plaque, and Conventional RFs for Long-Term Plaque Progression

	Model 1	Model 2		Model 3		
	Conventional RF	Conventional RF + Baseline Plaque		Conventional RF + Baseline Plaque + Polygenic Risk		
	AUC (95% CI)	AUC (95% CI)	*P* Value vs Model 1	AUC (95% CI)	*P* Value vs Model 1	*P* Value vs Model 2
PAV progression	0.74 (0.68–0.79)	0.83 (0.78–0.87)	<0.001	0.86 (0.81–0.90)	<0.001	0.053
NCPV progression	0.68 (0.62–0.74)	0.69 (0.63–0.75)	0.629	0.73 (0.67–0.79)	0.030	0.039

Shown are the areas under the curve (AUCs) of logistic regression models for prediction of above-median progression of PAV and percent noncalcified plaque volume (NCPV). Model 1 consisted of conventional risk factors only. Model 2 consisted of conventional risk factors and baseline PAV and NCPV, respectively. Model 3 consisted of conventional risk factors (RFs), baseline plaque, and PRS.

Abbreviations as in Tables 1 and 4.

## PERSPECTIVES

### COMPETENCY IN MEDICAL KNOWLEDGE:

In patients suspected of having CAD, polygenic risk is strongly associated with accelerated plaque progression and increased prevalence of HRPs over long-term follow-up.

### COMPETENCY IN PATIENT CARE AND PROCEDURAL SKILLS:

In patients with significant plaque burden, CAD PRS should be considered as a driver of progression of plaque and outcomes.

### TRANSLATIONAL OUTLOOK:

Prospective (randomized controlled) trials are required to assess whether routine inclusion of CAD PRS in conventional risk assessment reduces the progression of CAD.

## ABBREVIATIONS AND ACRONYMS

ASCVD: atherosclerotic cardiovascular disease
AUC: area under the curve
CACS: coronary artery calcium scoring
CAD: coronary artery disease
CTA: computed tomography angiography
HRP: high-risk plaque
LDL: low-density lipoprotein
MACE: major adverse cardiovascular events
PAV: percent atheroma volume
PR: positive remodeling
PRS: polygenic risk score
