# Supplementary material for: Polygenic Risk Is Associated With Long-Term Coronary Plaque Progression and High-Risk Plaque
Source: JACC Cardiovasc Imaging. Author manuscript; Available in PMC 2025 Jun 25. (PMC12190865; doi:10.1016/j.jcmg.2024.06.015)
Supplement: supplementary methods [file NIHMS2091574-supplement-supplementary_methods.pdf]

Supplemental Methods

Genotyping

DNA was genotyped using a customized Illumina Global Screening Array (GSA) v3 genotype array.[1] Sample quality control (calling missingness >5%, heterozygosity rate >5 standard deviations above the average, and mismatch between genotypically-determined and self-reported sex) did not lead to exclusion of individuals. Variant-level quality control was performed to remove those with a minor allele frequency of < 0.01 and those that were not in Hardy-Weinberg equilibrium in unrelated samples. Imputation of additional variants needed for PRS assessment was performed using the 1000 Genome Study phase 3 dataset as a reference panel.[2]

Polygenic risk score calculation

A previously published CAD PRS (GPS_{Mult}) of ~1.2 million common DNA variants was calculated in all individuals. This score was derived from genome-wide association data across five ancestries for CAD (>269,000 cases and >1,178,000 controls) and ten CAD risk factors.[3] In brief, a range of tuning parameters within the LDPred2 algorithm were used to select the best-performing polygenic score among 116,649 individuals (4,412 CAD cases and 112,237 controls) of European ancestry in the UK Biobank study.[3] Among these individuals, the GPS_{Mult} associated with an odds ratio for CAD per standard deviation increase of 2.14 (95% CI: 2.10-2.19; p<0.001).[3] GPS_{Mult} was additionally validated in several independent external datasets with >190,000 participants of African, European, Hispanic and South Asian ancestry, where it outperformed all available previously published CAD polygenic scores.[3] The polygenic score weights for GPS_{Mult} used in this publication are available for download from the Polygenic Score Catalog through accession ID PGS003725

(https://www.pgscatalog.org/score/PGS003725/). The raw calculated polygenic score was ancestry-adjusted and normalized using the first 10 principal components of genetic ancestry as performed previously.[3,4]

CCTA imaging

At baseline imaging, all patients underwent combined coronary artery calcium scoring (CACS) and CCTA using ≥64 slice CCTA scanners from the same manufacturer (Gemini TF 64 PET/CT and 256-slice Brilliance iCT; Philips Healthcare, Best, the Netherlands), as described previously.[5,6] Patients were administered sublingual nitroglycerin and, if necessary, metoprolol to achieve a heart rate below 65 beats per minute. First, CACS was obtained using a noncontrast computed tomography (CT) scan. Subsequently, CCTA was performed using a 120 kV tube voltage and a prospective electrocardiogram-gated CCTA protocol triggered at 75% of the R-R interval and with automatic tracking of 100 ml iobitridol bolus (Xenetix 350, Guerbet Nederland B.V., Gorinchem, the Netherlands). At follow-up, patients also underwent combined CACS and CCTA using a third-generation dual source CT scanner (SOMATOM Force, Siemens Healthineers, Germany). Patients were administered sublingual nitroglycerin and, if necessary, metoprolol to achieve a heart rate below 65 beats per minute. CCTA was performed with automated tube voltage and tube current modulation (CAREKv, CAREDose 4D, Siemens Healthineers, Germany) using a prospective electrocardiogram-gated CCTA protocol triggered at 70% of the R-R interval. CCTA was performed using a weight and kV dependent contrast dose (Xenetix 350, Guerbet Nederland B.V., Gorinchem, the Netherlands) after a test bolus.[7]

AI-QCT analysis

An artificial intelligence-based software approach was used to analyze the CCTA images (Atherosclerosis Imaging Quantitative Computed Tomography; AI-QCT; Cleerly Inc., Denver, CO).[8] This FDA-cleared software service utilizes a series of validated convolutional neural networks for image quality assessment, coronary segmentation and labeling, lumen wall evaluation, vessel contour determination, and plaque characterization. Prior validation of AI-QCT has been reported in multicenter trials vs expert consensus, quantitative coronary angiography and fractional flow reserve as previously published[8–10] as well as intravascular ultrasound.[11] The algorithm first produces a coronary centerline, lumen and outer vessel wall contouring for every phase available and subsequently selects the two most optimal series for analysis. The choice for best quality image is then made on a per-vessel basis. After automated segmentation and labeling in all vessels, plaques are characterized and quantified based on the Hounsfield unit (HU) attenuation. Finally, a trained radiologic technologist provides quality assurance overview of the AI analysis. AI-QCT and quality assurance was performed blinded for the PRS and clinical characteristics.

Coronary segments with a diameter ≥1.5mm were included in the analysis using the modified 18-segment SCCT model.[12] Coronary percentage stenosis was adjudicated on a per-vessel basis as per SCCT guidelines and categorized by the Coronary Artery Disease Reporting and Data System (CAD-RADS).[13] Each segment was evaluated for the presence or absence of coronary atherosclerosis, defined as any tissue structure >1 mm^2 within the coronary artery wall that was differentiated from the surrounding epicardial tissue, epicardial fat or the vessel lumen itself. Plaque volumes (mm^3) were calculated for each coronary lesion and then summated to compute the total plaque volume at the segment, vessel and patient level. At baseline, plaque volume was categorized using Hounsfield unit (HU) ranges, with low-density non-calcified plaque (LD-NCP) defined as plaques with any component on a

pixel level basis and quantified on an increment of 0.1 mm^3 as <30 HU, non-calcified plaque volume (NCPV) defined as HU between 30 and 350, and calcified plaque volume (CPV) defined as >350 HU.[14] To account for inevitable differences in scanner type and settings between baseline and follow-up due to the 10-year progression analysis, the plaque HU thresholds were adjusted for the different scanners and kV settings based on the luminal attenuation, to enable longitudinal comparison.[15]

Supplemental Figure 1. Flowchart of patient inclusion

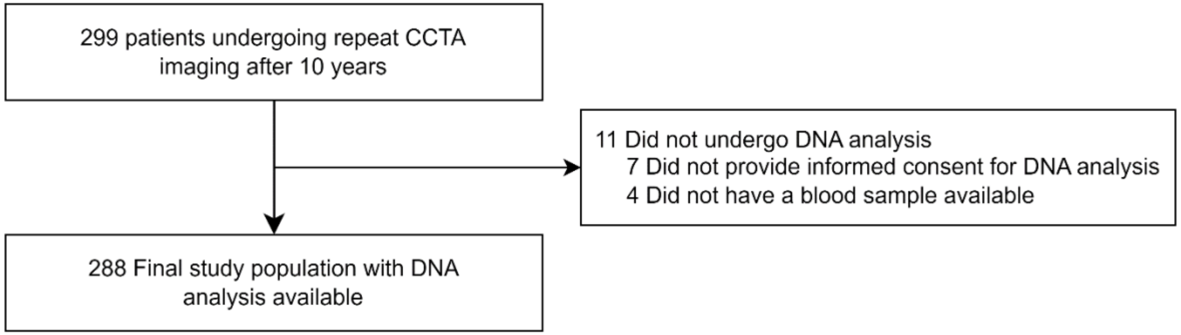

Flowchart of patient inclusion. CABG, coronary artery bypass grafting; CCTA, coronary CT angiography; GSA, Global Screening Assay.

Supplemental Table 1. Association of CAD PRS with baseline plaque volumes on a per-vessel level

	**Model 1** Unadjusted		**Model 2** Adjusted for age, sex, genetic ancestry, conventional risk factors	
	**β (95% CI)**	**P-value**	**β (95% CI)**	**P-value**
**Baseline percent atheroma volume (PAV)**				
CAD PRS, per 1SD increase	1.01 (0.26-1.77)	0.009	0.82 (0.11-1.53)	0.024
Genetic risk groups				
Low PRS	Ref	Ref	Ref	Ref
Intermediate PRS	0.43 (−1.48, 2.33)	0.662	−0.04 (−1.85, 1.77)	0.903
High PRS	3.73 (1.38, 6.08)	0.002	2.61 (0.37, 4.86)	0.023
**Baseline percent non-calcified plaque volume**				
CAD PRS, per 1SD increase	0.51 (0.06, 0.97)	0.028	0.41 (-0.02, 0.84)	0.065
Genetic risk groups				
Low PRS	Ref	Ref	Ref	Ref
Intermediate PRS	0.21 (−0.94, 1.37)	0.715	−0.07 (−1.18, 1.04)	0.899
High PRS	1.92 (0.50, 3.34)	0.008	1.35 (−0.02, 2.72)	0.055
**Baseline percent calcified plaque volume**				

CAD PRS, per 1SD increase	0.49 (0.15,0.84)	0.006	0.40 (0.08,0.73)	0.016
Genetic risk groups				
Low PRS	Ref	Ref	Ref	Ref
Intermediate PRS	0.23 (−0.64, 1.11)	0.601	0.08 (−0.75, 0.92)	0.842
High PRS	1.75 (0.67, 2.84)	0.002	1.22 (0.19, 2.26)	0.021

Unadjusted and adjusted mixed effect models were constructed with baseline percent atheroma volume, baseline percent non-calcified plaque volume and baseline percent calcified plaque volume as dependent outcomes using both CAD PRS on a continuous scale and divided in the different genetic risk groups on a per-vessel level. The multivariable model was adjusted for age, sex, genetic ancestry, coronary revascularization, and conventional risk factors (hypertension, hypercholesterolemia, diabetes, body mass index, smoking status, family history of CAD and statin use). CAD, coronary artery disease; PRS, polygenic risk score.

Supplemental Table 2. Changes in plaque volumes between baseline and 10-year follow-up adjusted for baseline plaque volumes

Characteristic	Polygenic risk				
	Low<br>N = 58	Intermediate<br>N = 172		High<br>N = 58	
		β (95% CI)	P-value	β (95% CI)	P-value
Percent atheroma volume (PAV)	Ref	1.54 (0.12, 2.97)	0.035	2.33 (0.56, 4.10)	0.010
Percent non-calcified plaque volume	Ref	1.07 (0.13, 2.01)	0.027	1.55 (0.39, 2.72)	0.009
Percent calcified plaque volume	Ref	0.98 (-0.37, 2.32)	0.156	1.73 (0.06, 3.40)	0.043

Shown are the β (95% CI) coefficients for progression of percent atheroma volume (PAV), percent non-calcified plaque volume and percent calcified plaque volume after adjustment for baseline plaque volumes in low, intermediate and high PRS groups. Vessels that were revascularized between baseline and follow-up were excluded from both baseline and follow-up plaque analysis. Data are shown as median (IQR) or n (%). PRS, polygenic risk score.

Supplemental Table 3. Association of CAD PRS with plaque progression over 10 years on a per-vessel level

	**Model 1** Unadjusted		**Model 2** Adjusted for age, sex, genetic ancestry, conventional risk factors and baseline plaque volume	
	**β (95% CI)**	**P-value**	**β (95% CI)**	**P-value**
**Percent atheroma volume (PAV), change per 10 years of follow-up**				
CAD PRS, per 1SD increase	0.87 (0.56-1.18)	<0.001	0.63 (0.33, 0.94)	<0.001
Genetic risk groups				
Low PRS	Ref	Ref	Ref	Ref
Intermediate PRS	1.40 (0.62, 2.18)	<0.001	0.71 (−0.07, 1.55)	0.074
High PRS	3.24 (2.25, 4.23)	<0.001	2.43 (1.44, 3.42)	<0.001
**Percent non-calcified plaque volume, change per 10 years of follow-up**				
CAD PRS, per 1SD increase	0.35 (0.13, 0.56)	0.001	0.27 (0.06, 0.49)	0.013
Genetic risk groups				
Low PRS	Ref	Ref	Ref	Ref
Intermediate PRS	1.03 (0.5, 1.56)	<0.001	0.87 (0.32, 1.41)	0.002
High PRS	1.19 (0.45, 1.87)	<0.001	0.92 (0.22, 1.62)	0.010
**Percent calcified plaque volume, change per 10 years of follow-up**				

CAD PRS, per 1SD increase	0.59 (0.39, 0.79)	<0.001	0.46 (0.27, 0.66)	<0.001
Genetic risk groups				
Low PRS	Ref	Ref	Ref	Ref
Intermediate PRS	0.61 (0.11, 1.10)	0.016	0.14 (−0.36, 0.63)	0.593
High PRS	2.15 (1.52, 2.78)	<0.001	1.73 (1.09, 2.36)	<0.001

Unadjusted and adjusted mixed effect models were constructed with percent atheroma volume, percent non-calcified plaque volume and percent calcified plaque volume as dependent outcomes on a per-vessel level. An interaction term between the duration of follow-up and CAD PRS (both continuous and the CAD PRS groups) and the other covariables was included to estimate plaque progression during follow-up. The multivariable model was adjusted for age, sex, genetic ancestry, coronary revascularization, and conventional risk factors (hypertension, hypercholesterolemia, diabetes, body mass index, smoking status, family history of CAD and statin use). CAD, coronary artery disease; PRS, polygenic risk score.

Supplemental Table 4. Association of CAD PRS with plaque progression over 10 years on a per-vessel level in statin-treated patients

	**Model 1** Unadjusted		**Model 2** Adjusted for age, sex, genetic ancestry, conventional risk factors and baseline plaque volume	
	**β (95% CI)**	**P-value**	**β (95% CI)**	**P-value**
**Percent atheroma volume (PAV), change per 10 years of follow-up**				
CAD PRS, per 1SD increase	0.60 (0.21, 0.99)	0.002	0.59 (0.20, 0.98)	0.003
Genetic risk groups				
Low PRS	Ref	Ref	Ref	Ref
Intermediate PRS	1.68 (0.63, 2.74)	0.002	0.68 (−0.41, 1.76)	0.222
High PRS	2.60 (1.34, 3.86)	<0.001	2.07 (0.8, 3.34)	0.001
**Percent non-calcified plaque volume, change per 10 years of follow-up**				
CAD PRS, per 1SD increase	0.33 (0.05, 0.60)	0.019	0.28 (0.01, 0.56)	0.045
Genetic risk groups				
Low PRS	Ref	Ref	Ref	Ref
Intermediate PRS	1.58 (0.84, 2.32)	<0.001	1.04 (0.26, 1.81)	0.009
High PRS	1.41 (0.53, 2.29)	0.002	1.06 (0.15, 1.96)	0.022
**Percent calcified plaque volume, change per 10 years of follow-up**				

CAD PRS, per 1SD increase	0.41 (0.17, 0.65)	<0.001	0.45 (0.21, 0.68)	<0.001
Genetic risk groups				
Low PRS	Ref	Ref	Ref	Ref
Intermediate PRS	0.56 (−0.09, 1.21)	0.093	0.11 (−0.56, 0.78)	0.742
High PRS	1.58 (0.80, 2.36)	<0.001	1.40 (0.62, 2.18)	<0.001

Unadjusted and adjusted mixed effect models were constructed with percent atheroma volume, percent non-calcified plaque volume and percent calcified plaque volume as dependent outcomes on a per-vessel level in patients using statins during follow-up (n=215). An interaction term between the duration of follow-up and CAD PRS (both continuous and the CAD PRS groups) and the other covariables was included to estimate plaque progression during follow-up. The multivariable model was adjusted for age, sex, genetic ancestry, coronary revascularization, and conventional risk factors (hypertension, hypercholesterolemia, diabetes, body mass index, smoking status, family history of CAD and statin use). CAD, coronary artery disease; PRS, polygenic risk score.

Supplemental References

1. Ibrahim S, van Rooij J, Verkerk AJMH, et al. Low-Cost High-Throughput Genotyping for Diagnosing Familial Hypercholesterolemia. *Circ Genomic Precis Med*. 2023;16(5):462-469. doi:10.1161/circgen.123.004103

2. Auton A, Abecasis GR, Altshuler DM, et al. A global reference for human genetic variation. *Nature*. 2015;526(7571):68-74. doi:10.1038/nature15393

3. Patel AP, Wang M, Ruan Y, et al. A multi-ancestry polygenic risk score improves risk prediction for coronary artery disease. *Nat Med*. 2023;29(7):1793-1803. doi:10.1038/s41591-023-02429-x

4. Wang M, Menon R, Mishra S, et al. Validation of a Genome-Wide Polygenic Score for Coronary Artery Disease in South Asians. *J Am Coll Cardiol*. 2020;76(6):703-714. doi:10.1016/j.jacc.2020.06.024

5. van Diemen PA, Bom MJ, Driessen RS, et al. Prognostic Value of RCA Pericoronary Adipose Tissue CT-Attenuation Beyond High-Risk Plaques, Plaque Volume, and Ischemia. *JACC Cardiovasc Imaging*. 2021;14(8):1598-1610. doi:10.1016/j.jcmg.2021.02.026

6. Nurmohamed NS, Bom MJ, Jukema RA, et al. AI-Guided Quantitative Plaque Staging Predicts Long-Term Cardiovascular Outcomes in Patients at Risk for Atherosclerotic CVD. *JACC Cardiovasc Imaging*. 2023. doi:https://doi.org/10.1016/j.jcmg.2023.05.020

7. van den Boogert TPW, Lopes RR, Lobe NHJ, et al. Patient-tailored Contrast Delivery Protocols for Computed Tomography Coronary Angiography: Lower Contrast Dose and Better Image Quality. *J Thorac Imaging*. 2021;36(6).

https://journals.lww.com/thoracicimaging/fulltext/2021/11000/patient_tailored_contrast_delivery_protocols_for.3.aspx.

8. Griffin WF, Choi AD, Riess JS, et al. AI Evaluation of Stenosis on Coronary CT Angiography, Comparison With Quantitative Coronary Angiography and Fractional Flow Reserve: A CREDENCE Trial Substudy. *JACC Cardiovasc Imaging*. 2022. doi:https://doi.org/10.1016/j.jcmg.2021.10.020

9. Jonas R, Earls J, Marques H, et al. Relationship of age, atherosclerosis and angiographic stenosis using artificial intelligence. *Open Hear*. 2021;8(2):e001832. doi:10.1136/openhrt-2021-001832

10. Choi AD, Marques H, Kumar V, et al. CT Evaluation by Artificial Intelligence for Atherosclerosis, Stenosis and Vascular Morphology (CLARIFY): A Multi-center, international study. *J Cardiovasc Comput Tomogr*. 2021;15(6):470-476. doi:10.1016/j.jcct.2021.05.004

11. Omori H, Matsuo H, Earls J, et al. Abstract 13665: Determination of Lipid-Rich Plaque by Artificial Intelligence-Enabled Quantitative Computed Tomography Using Near-Infrared Spectroscopy. *Circulation*. 2022;146(Suppl_1):A13665-A13665. doi:10.1161/circ.146.suppl_1.13665

12. Leipsic J, Abbara S, Achenbach S, et al. SCCT guidelines for the interpretation and reporting of coronary CT angiography: A report of the Society of Cardiovascular Computed Tomography Guidelines Committee. *J Cardiovasc Comput Tomogr*. 2014;8(5):342-358. doi:10.1016/j.jcct.2014.07.003

13. Cury RC, Blankstein R, Leipsic J, et al. CAD-RADS™ 2.0 - 2022 Coronary Artery Disease – Reporting and Data System an expert consensus document of the Society of Cardiovascular Computed Tomography (SCCT), the American College of Cardiology

(ACC), the American College of Radiology (ACR) and the No. *J Cardiovasc Comput Tomogr*. 2022;16(6):536-557. doi:10.1016/j.jcct.2022.07.002

14. Shaw LJ, Blankstein R, Bax JJ, et al. Society of Cardiovascular Computed Tomography / North American Society of Cardiovascular Imaging – Expert Consensus Document on Coronary CT Imaging of Atherosclerotic Plaque. *J Cardiovasc Comput Tomogr*. 2021;15(2):93-109. doi:10.1016/j.jcct.2020.11.002

15. Dey D, Schepis T, Marwan M, Slomka PJ, Berman DS, Achenbach S. Automated three-dimensional quantification of noncalcified coronary plaque from coronary CT angiography: Comparison with intravascular US. *Radiology*. 2010;257(2):516-522. doi:10.1148/radiol.10100681
